# Association of Beta-Thalassaemia and Hypogonadotropic Hypogonadism

**DOI:** 10.1155/2022/4655249

**Published:** 2022-05-19

**Authors:** Angela Vidal, Carolin Dhakal

**Affiliations:** Department of Obstetrics and Gynecology, Lucerne Cantonal Hospital, Switzerland

## Abstract

Thalassaemic syndromes are among the most common haemoglobinopathies and are associated with high morbidity and mortality. Because of the various treatments, a secondary endocrinopathy due to iron overload—haemosiderosis—can occur, causing hypopituitarism leading to hypogonadotropic hypogonadism (HH) and infertility. We present a case of secondary amenorrhoea in a patient with beta-thalassaemia and a history of multiple therapies in her adolescence, such as multiple transfusions, chemotherapy, and allogeneic bone marrow transplantation, who presented with HH and premature ovarian insufficiency.

## 1. Background

The global incidence of haemoglobinopathy is 7%. Thalassa syndrome is one of the most common haemoglobinopathies associated with high morbidity and mortality rates [1]. Beta-thalassaemia is a severe haematological disease that results in reduced haemoglobin synthesis due to a genetic component [2]. Patients without transfusions experience massive erythroid expansion and severe haemolytic anaemia. Iron overload is a consequence of frequent blood transfusions, which is the most important treatment modality for thalassaemia major. Such treatment is the cause of secondary endocrinopathy due to iron overload, haemosiderosis, causing hypopituitarism, which leads to hypogonadotropic hypogonadism (HH). Other possible causes of hypogonadism in beta-thalassaemia major are liver disorders, chronic hypoxia, diabetes mellitus, and zinc deficiency [3, 4].

The quality of life of thalassaemic patients is affected by the need for chronic transfusions, lifelong iron chelation therapy, and related complications. Allogeneic haematopoietic stem cell transplantation (allo-HSCT) is a potentially curative treatment for transfusion-dependent patients without iron-related complications, especially at a young age. In some cases, bone marrow transplants are not sex-matched [5, 6].

Beta-thalassaemia major and its possible treatments may cause premature ovarian insufficiency (POI). POI is one of the leading causes of infertility in women worldwide. POI is defined as the presence of amenorrhoea for at least four consecutive months in women under 40 years of age and twice elevated follicle − stimulating hormone (FSH) levels > 25 IU/L according to the European Society for Human Reproduction and Embryology (ESHRE) guideline 2015 [7]. POI affects 1%–1.2% of the general population, and pregnancy with POI is rare (<1 : 9200). The following are the causes of POI: prior ovarian surgery, chemotherapy or radiation therapy, personal or family history of autoimmune diseases, family history of POI (fragile X premutation), or other genetic reasons [7, 8].

We present a case of secondary amenorrhoea in a patient with beta-thalassaemia and a history of multiple therapies in her adolescence, such as multiple transfusions, chemotherapy, and allogeneic bone marrow transplantation, presenting with HH and POI.

We discuss possible fertility problems after different treatments due to beta-thalassaemia.

### 1.1. Case Report

A 38-year-old woman was referred to our clinic with a 5-year history of amenorrhoea and infertility. Important diagnoses in her medical history are a beta-thalassaemia major since childhood and treatment with periodic blood transfusions to maintain adequate haemoglobin levels. From the age of 10 years, she was diagnosed with restrictive heart disease and treated with digoxin. She was also diagnosed with fibrosis of the liver, siderosis, and fibrosis of the pancreas. At the age of 19 years, she underwent a bone marrow transplant, at which time she underwent pretreatment with fludarabine, busulfan, and cyclophosphamide for 15 days as adjuvant chemotherapy. Her human leukocyte antigen- (HLA-) identical donor was her brother.

Her menarche was at 13 years of age. She had a regular menstrual cycle until she was 15 years old. Functional hypothalamic amenorrhoea was suspected due to the patient's medical history. Other possible causes of functional hypothalamic amenorrhoea, such as eating disorders, excessive exercise, and stress, were ruled out.

The laboratory test results showed reduced levels of FSH (1.06 IU/L), luteinizing hormone (LH) (<0.3 IU/L), oestradiol (>18.4 pmol/L), and antimullerian *hormone* (AMH) (<0.007 pmol/L). A gonadotropin-releasing *hormone* (GnRH)/luteinizing *hormone*-releasing *hormone* (LHRH) test was performed with a 0.1 mg synthetic LHRH bolus administered intravenously. Serum samples were extracted for gonadotropin measurements at 0 min (FSH 1.03 IU/L and LH < 0.3 IU/L) and 30 min after LHRH administration (FSH 1.15 IU/L and LH 0.57 IU/L). Even after LHRH stimulation, the pituitary response was subnormal, consistent with HH. A stimulation test with human menopausal gonadotropin 225 IE was performed without any detectable response.

**Figure 1:**
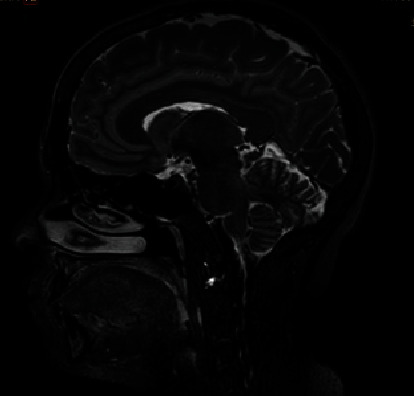
MRI of the pituitary gland, which is rather small for age and sex but without evidence of focal abnormalities.

Peak levels of growth hormone and cortisol with the insulin tolerance test were 11.6 ng/mL and 26.3 *μ*g/dL, respectively. Magnetic resonance imaging (MRI) findings showed a decrease in the intensity of the pituitary gland signal on T2-weighted and size images (Figure 1).

Based on the above characteristics, the patient was diagnosed with secondary amenorrhoea and POI.

The patient was referred to a fertility centre abroad for oocyte donation, as this treatment option is not permitted in Switzerland. She became pregnant through oocyte donation.

The pregnancy and delivery ensued without complications. A primary caesarean section was performed due to maternal risk of complications during delivery. The patient delivered a boy with a normal weight of 3180 g, with normal pH and Apgar scores. Six weeks postpartum after cessation of voluntary breastfeeding, she was started on hormone replacement therapy with oestradiol and norethisterone because of persistent amenorrhoea due to POI. *Hormone replacement* therapy was recommended until the onset of normal menopause at 51 years of age.

## 2. Discussion

The differential diagnosis in patients with secondary amenorrhoea diagnosed with beta thalassaemia and after receiving various treatments is of great relevance due to the possible management and treatment depending on the aetiology. Here, we describe the possible causes:

- Primary ovarian insufficiency (POI)

POI is condition that affects 1 in 100 women under the age of 40 years (1%), 1 in 1000 women under the age of 30 years (0.1%), and 1 in 10,000 women under the age of 20 years (0.01%) [7, 9]. According to the ESHRE, POI is a clinical syndrome defined by the loss of ovarian activity before the age of 40 years. POI is characterised by menstrual disturbance (amenorrhoea) with increased gonadotropins and low oestradiol levels [7].

Although the exact cause is unknown in most cases, some of the causes of POI have been identified, including genetic disorders (such as fragile X syndrome and Turner syndrome, where genetic testing should be performed), autoimmune diseases (such as thyroiditis and Addison's disease), and iatrogenic causes (such as chemotherapy or other gonadotoxic treatments) [7, 8].

As a consequence of allogeneic bone marrow transplantation, it must be taken into account when carrying out genetic tests to ensure that the DNA mutations detected come from the patient's native DNA; therefore, the most suitable and reliable biological sample for DNA isolation must be obtained, and fibroblast culture is recommended [10].

(ii) Hypothalamic and pituitary causes

*Functional hypothalamic amenorrhoea*. Functional hypothalamic amenorrhoea is a chronic anovulation disorder caused by suppression of the hypothalamic–pituitary axis due to body weight loss, eating disorders, excessive exercise, stress, or iron overload and may result in infertility or loss of bone density [11].

(iii) Other central nervous system causes

Secondary amenorrhoea may be caused by damage to the hypothalamic–pituitary axis due to inflammation, ischemia, infiltration, infection, iron overload, or trauma. Disorders affecting pubertal development, such as gonadotropin-releasing hormone deficiency and constitutional delay, may cause secondary amenorrhoea [12].

Beta-thalassaemia is a severe transfusion-dependent anaemia that causes infertility due to iron deposition in the endocrine organs, especially after overtransfusion. This deposition is a major cause of POI. Most patients are infertile due to HH and require assisted reproductive techniques. Studies have been conducted in female patients with beta-thalassaemia to investigate the hypothalamic–pituitary–ovarian axis [13–15]. In the thalassaemic group, basal and peak posttest levels of GnRH and other reproductive hormones were significantly lower than those in the control group. Iron-induced effects play a central role in the pathogenesis of decreased reproductive capacity.

There are different treatment approaches for individuals with different types of beta-thalassaemia, including bone marrow transplantation, blood transfusion, and chelation. The choice of treatment depends on the patient's age, severity of symptoms, and response to treatment. Blood transfusion is the main treatment for individuals with moderate to severe thalassaemia [16].

Frequent blood transfusions can cause infertility in adolescents. Infertility is often due to HH, which is a consequence of transfusion haemosiderosis. Excessive deposition of iron and oxidative iron compounds occurs with blood transfusion, leading to dysfunction of the female reproductive axis [17].

Bone marrow and blood transplantation are also used for the treatment of beta-thalassaemia. Bone marrow and blood transplantation allow the replacement of defective stem cells with healthy stem cells from a compatible donor. Thus, this is the most effective treatment method. However, it is difficult to find a good donor match for a small number of patients with severe thalassaemia.

Thirty to eighty percent of patients with transfusion-dependent thalassaemia show long-term ovulatory dysfunction and hypogonadism. Iron chelation therapy is also a mode of treatment for beta-thalassaemia [18, 19]. This treatment approach is mainly due to regular blood transfusion [13]. Regular blood transfusion leads to excessive accumulation of iron in the body, causing iron overload. Iron overload can cause infertility and damage to the liver and heart.

HSCT is a well-established and often the only curative treatment for severe haematological diseases. Therefore, the number of stem cell transplantations for nonmalignant diseases has continuously increased over the last decades and has led to an overall improvement in the survival of patients with congenital and acquired nonmalignant diseases [20, 21].

There is already much information in the literature on fertility preservation in patients with cancer. However, there are other pathologies, such as haematological nonmalignant diseases, where there are no clear clinical guidelines for fertility preservation [20, 22].

All prepubertal and postpubertal patients should be counselled on the options for fertility preservation measures that may be appropriate for them. Counselling should include information on the risk of diminished fertility—as a result of the intended treatment in relation to disease, age, previous therapy, and other comorbidities. The concept of fertility preservation is an integral part of treatment. During counselling, the possibility of inheritance of the underlying disease and possible pregnancy complications should be addressed [20].

Various fertility preservation options are available, such as oocyte cryopreservation, embryo cryopreservation, ovarian tissue transplantation, or ovarian function suppression by GnRH-analogues [21].

A 2019 publication describes the best methods to assess ovarian reserve after chemotherapy or total body irradiation, such as dynamic levels of FSH and oestradiol. It should be noted that antral follicle count and AMH levels are menstrual cycle-independent markers of ovarian reserve. In the case of beta-thalassaemia with haemosiderosis due to multiple transfusions, there may be iron-induced damage to the pituitary gland and/or ovarian tissue [23].

Currently, there are several nongonadotoxic therapeutic approaches that may open up a new pathway for beta-thalassaemia, such as preservation of hormonal function and fertility [24].

Future treatments for beta-thalassaemia depend on the benefit/risk/cost ratio of conventional transfusion, iron chelation, allogeneic bone marrow transplantation, and gene therapy, as well as the availability of these treatments in different health systems.

Gene therapy is still at an early stage of development, but several clinical studies have demonstrated the principle of sustained clinical efficacy with low toxicity in a number of patients [24].

Until such therapies are established, there is a high risk of POI due to the various beta-thalassaemia therapies; therefore, a detailed assessment of fertility preservation in adolescents and young adults should be undertaken if a multidisciplinary approach is adopted.

In our clinical case, the cause of POI would be the absence of fertility treatment prior to cyclophosphamide exposure.

The incidence of temporary amenorrhoea or early menopause following chemotherapy or radiotherapy varies with age, existing ovarian reserve, type of chemotherapy, and cumulative chemotherapy dose. Cyclophosphamide causes progressive and irreversible damage to oocytes in a dose-dependent manner. Chemotherapy with alkylating agents, such as cyclophosphamide, is associated with the highest risk of amenorrhoea [24].

In our case report, genetic testing (karyotype and fragile X syndrome) was performed to rule out genetic causes of POI. The karyotype revealed a 46,XY karyotype due to the bone marrow transplant of her HLA-identical brother. The XX karyotype was not verified. Quantitative fluorescent PCR analysis was performed, which resulted in a mixture of the two karyotypes. Low levels of gonadotrophins may obscure a POI in patients with severe beta-thalassaemia major. Due to the undetected ovarian reserve, a diagnosis of POI was made.

In conclusion, the main cause of infertility in patients with beta-thalassaemia major is HH. Endocrine complications occur due to iron overload secondary to frequent blood transfusions and bone marrow transplantation (resulting in hypergonadotropic hypogonadism). The treatment of infertility in patients with thalassaemia involves thorough counselling and fertility preservation before therapy. Nongonadotoxic alternative therapies, such as gene therapy, may be a safe alternative for such patients.

## Data Availability

The data that support the findings of this study are available from the corresponding author upon reasonable request.
